# Resistance and Strength of Conductive PLA Processed by FDM Additive Manufacturing

**DOI:** 10.3390/polym14040678

**Published:** 2022-02-10

**Authors:** Juraj Beniak, Ľubomír Šooš, Peter Križan, Miloš Matúš, Vít Ruprich

**Affiliations:** 1 Faculty of Mechanical Engineering, Slovak University of Technology in Bratislava, Nam. Slobody 17, 812 31 Bratislava, Slovakia; lubomir.soos@stuba.sk (Ľ.Š.); peter.krizan@stuba.sk (P.K.); milos.matus@stuba.sk (M.M.); 2 4machines s.r.o., Studentská 6202/17, 708 00 Ostrava-Poruba, Czech Republic; 4machines@4machines.cz

**Keywords:** fused deposition modeling, FDM, additive manufacturing, conductive, electro conductive, resistance, strength

## Abstract

There is a large number of materials that can be used for FDM additive manufacturing technology. These materials have different strength properties, they are designed for different purposes. They can be highly strong or flexible, abrasion-resistant, or designed for example for environments with higher thermal loads. However recently new innovative and progressive materials have come to the practice, which include nano-composite particles, bringing new added value. One such material is the Conductive PLA material, which is capable of conducting electric current. The aim of this article is to present the material properties of this material. The article describes the design of the experiment, the process of measuring the resistance of samples printed by FDM device, measuring the maximum tensile strength of samples. The article includes a statistical evaluation of the measured data, with the determination of the significance of individual factors of the experiment as well as the evaluation of the overall result of the experiments.

## 1. Introduction

Fused Deposition Modeling (FDM) technology is just one from many technologies which are covered with Additive Manufacturing (AM) process. It is possible to produce components from metallic materials, from resins and also from polymers [1,2,3]. Fused Deposition Modeling is the most wide-spread technology. Is very simple and also not so expensive as others. Using plastic wire as input material which is semi-melted and then by nozzle is deposited layer by layer to the required shape, defined by 3D digital mode [4,5,6]. Very often are used materials as PLA (polylactic acid), ABS (Acrylonitrile butadiene styrene), PETG (Polyethylene terephthalate glycol), Nylon, PC (Polycarbonate) or many others [7,8,9].

As composite materials we can see for example wood composites, where in basic polymer (mentioned above) are mixed wood particles. There is mostly up to 40% of wood particles [10].

The same way are produced also the composites with brass particles, copper particles, bronze particles and others. Modern and innovative filaments with progressive properties are created with nanoparticles of different composition [11,12,13,14,15].

The following article is focused on experimental investigation of the properties of the material Conductive PLA, which is designed for Additive Production using FDM (Fused Deposition Modeling) technology. This is material from Protopasta company (Vancouver, WA, USA). It consists of a basic matrix of thermoplastic Naturework 4043D PLA (Polylactic acid). The base matrix contains particles of Carbon Black material, which provides a specific property, electro-conductivity. Electro—conductive composite materials attract considerable industry attention, especially for their wide range of applications. By adding conductive fillers to the base matrix, it is an effective way to achieve such exceptional polymer properties. Carbon Black is now widely used in industry due to its low price, low weight and wide and easy availability [16,17,18]. To achieve conductivity, it is necessary to use a relatively high content of carbon black in the base material, which can have a significant effect on strength, flexibility, but also other material properties [19,20,21].

**Figure 1:**
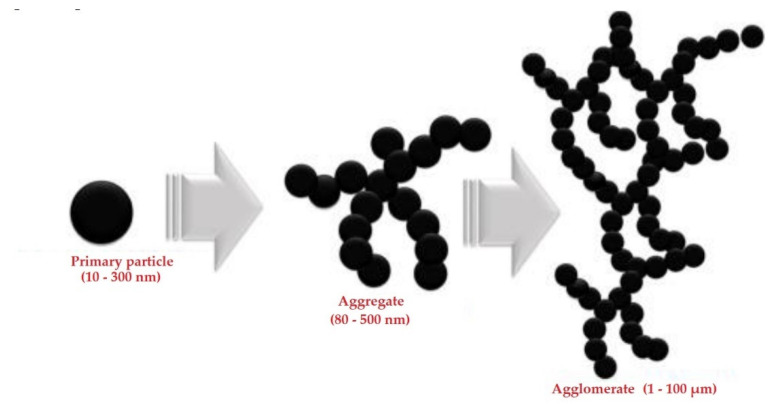
Carbon particles formed to aggregates in bulk phase. Reproduced from [36] with permission from Elsevier 2019.

Carbon partcles could be used in such applications basically in three types of carbon fillers. Carbon Black (CB), Carbon Fiber (CF) and Carbon Nano-Tubes (CNT) [22]. They are used as fillers in basic polymers. Can be used alone or in combinations. Carbon fibers have been widely used recently, mainly because of their greater availability, unlike carbon nanotubes, which are also more expensive and less affordable [23,24,25]. Different structures, morphology and shapes of these fillers, their dispersion and other properties affect the conductivity of the prepared materials [26,27,28,29,30,31]. Carbon Black is the most commonly used filler due to its low cost, low density and good internal conductivity. Some studies suggest that the morphology of CB aggregates in a polymer matrix is grape shape like (Figure 1), consisting of many individual CB particles with an average diameter of tens of nanometers [32,33,34,35].

**Figure 2:**
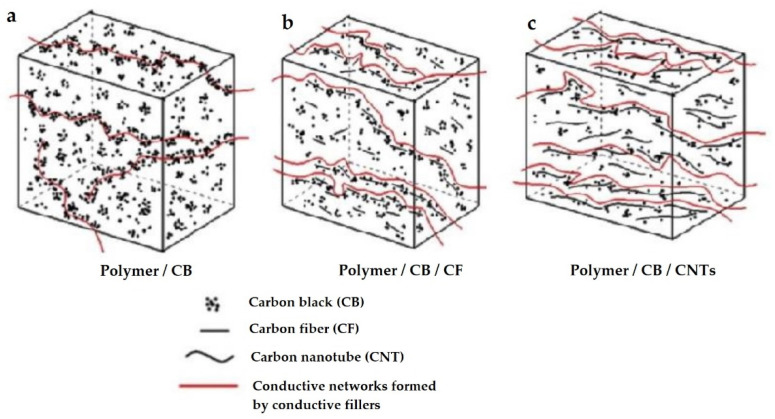
The conductive path formed by Carbon Black (**a**), Carbon black and Carbon fiber (**b**), Carbon Black and Carbon Nanotubes (**c**). Reproduced from [37] with permission from Elsevier 2012.

The size of the aggregates and the distance between them are crucial for creating electrical conductivity [36,37,38,39]. The distances between the nearest adjacent multiparticulate surfaces must be narrow enough to create suitable conditions for the passage of electric current. The average inter-particle distance should be from 10 to 28 nm [15]. Figure 2 shows 3 types of Carbon Black, Carbon fibers and Carbon nanotubes.

Such a material as filler could be used within basic thermoplastic mentioned above. When using the fillers, material properties are changed. Increasing the Carbon Black content within the matrix increasing the conductivity but on the other hand decreasing the strength of final product. Following part of this paper will deal with experimental determination of material properties of Conductive PLA material.

## 2. Materials and Methods

As mentioned above, the experimentally tested material is Conductive PLA. This material consist of Naturework 4043D PLA (Vancouver, WA, USA), thermoplastic and Carbon Black particles. The weight ratio of carbon black is about 25%. Material is in filament form, and is quite flexible, and is compatable with any PLA printing printer. Based on information from producer, Protopasta Conductive PLA is a good choice for low-voltage circuitry applications, touch sensor projects, and using prints to interact with touch screens, which require low conductivity.

The other resistance properties stated by producer are:

- Volume resistivity of molded resin (not 3D Printed): 15 ohm-cm
- Volume resistivity of 3D printed parts perpendicular to layers: 30 ohm-cm
- Volume resistivity of 3D printed parts through layers (along Z axis): 115 ohm-cm
- Resistance of a 10 cm length of 1.75 mm filament: 2–3 kohm
- Resistance of a 10 cm length of 2.85 mm filament: 800–1200 ohm

Filament is in 1.75 mm diameter, supplied on spool. Other setting of 3D printer is very similar to conventional PLA filament.

As mentioned above, there will be two types of experiment. One is for tensile strength testing. Second for testing of resistance with different influencing factors change.

### 2.1. Tensile Strength Measurement

**Figure 3:**
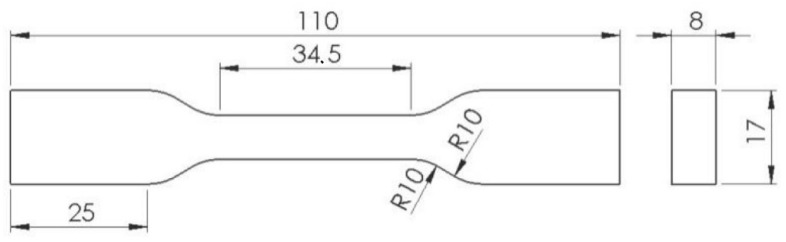
Specimens for tensile strength testing.

For tensile strength measurement, there were produced testing specimens (Figure 3), designed with following of ISO standards ISO 527-1.

Before the specimens were produced on FDM 3D printer, the design of experiment is prepared. First of all factors had to be stated. We selected the type of infill, to figure out which is better for this kind of material. Selected are Rectilinear and Honeycomb as the most used in the practice. Then we specified two layer thickness for producing od specimens. Selected are 0.125 mm and 0.25 mm. The last specified factor is infill volume. We selected 50% to figure out, how the decreasing of volume change the measured tensile strength. The second level of infill volume is 90%, because 100% is not possible with honeycomb infill. But for comparison we produced also the 100% infill, but not with all of others combinations. Selected factors and their levels are specified in (Table 1). Full factor experiment have been prepared to take into the consideration all the combinations of factors and levels (Table 2).

**Figure 4:**
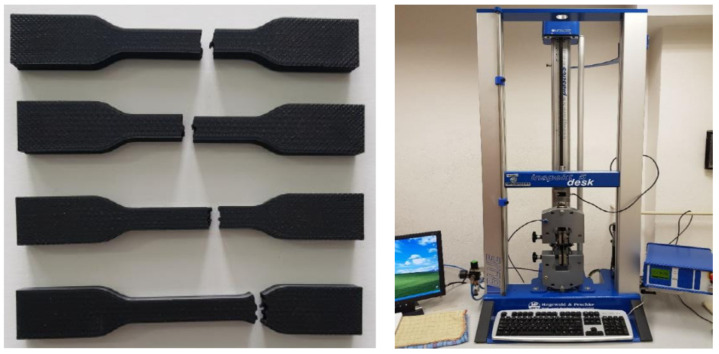
Specimens and testing device for experiment.

Based on described information, the specimens are produced. There have been produced 4 specimens with the same settings based on defined design of experiment. After that, all produced specimens were tested on universal testing device Inspekt Desk 5 kN (Hegewald & Peschke, Nossen, Germany) (Figure 4). The maximum testing force for this device is 5 thousand newton.

### 2.2. Resistance Measurement

The primary goal conductive material experiment is to determine the resistance of this material. In this case we want to first of all figure out how is the resistance changing with length of produced samples. So length is the first factor for this experiment. We decided to produce specimens from the minimum length 10 mm, then 20 mm and with this 10 mm differentiation up to length 100 mm.

The second factor is printing nozzle temperature. The purpose if to figure out how the printing temperature affecting final resistance. If the more molted and deposited material have better or worst resistance. Depending on minimum and maximum printing temperature advised by producer, we selected 190 °C and 220 °C.

The last factor is temperature during the resistance measurement process. One measurement were made within normal room temperature 25 °C, and the second with much higher temperature 80 °C.

Selected factors and their levels are presented in Table 3.

Based on this selected factors and levels, the full factor is prepared, with all of the combinations as is shown on Table 4. For each combination 5 samples were produced. So each experiment is repeated 5 times.

**Figure 5:**
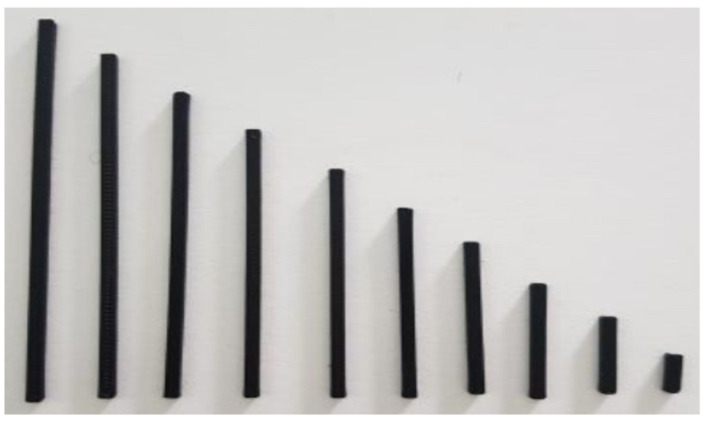
Specimens for resistance measurement, length from 10 mm to 100 mm.

For this purpose are produced very simple specimens (Figure 5) with square cross-section and with specified length as defined in design of experiment. For measurement is used conventional digital multimeter PU510 with accuracy ±0.5%. Each measurement were repeated five time, to prevent some random errors.

## 3. Results and Discussion

### 3.1. Evaluation of Tensile Strength Measurement

For each setup (experiment) 4 samples were measured, for repeatability of measurements and exclusion of random errors. A total of 32 measurements were performed. Measured values are placed to the Table 5, where is also visible the design of experiment for better orientation and recognizing which values belong to which combination of factors and their levels.

**Figure 6:**
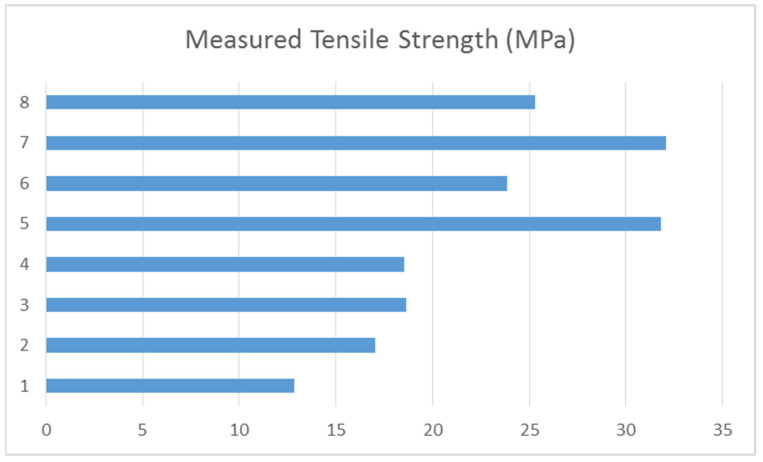
Presentation of measured tensile strength for each experiment.

All average values from each measurement and each experiment are also placed to the Figure 6, where are graphically displayed. There is better visualized differences among of single measurements. The highest values of tensile strength belong to the experiments 5 and 7. Both of them belong to the second part of experiments (5 to 8) with highest measured values. It is pretty logical, because the only difference is infill volume. If we are increasing the infill volume it will naturally express to the higher strength.

**Figure 7:**
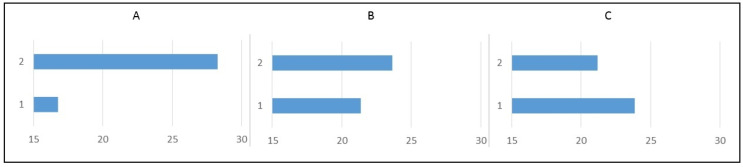
Graphical presentation of factors weights to measured tensile strength. (**A**)—Length, (**B**)—Nozzle Temperature, (**C**)—Measurement temperature.

For very fast and easy evaluation we just made weight comparison of each factors with its levels (Figure 7). From the graphical expression is possible to see that the bigger influence comes from factor A, which is presented by infill volume. As we stated above, it is naturally and expected. The others two factor have significant influence, but not so big as factor A.

For exact evaluation of measured values and exact statement if some factor is significant or not, the ANOVA statistical method have been executed. For this purpose is used software for easier and faster evaluation. The final output from the software is visible in below (Table 6). There are mentioned all of the factors and also all interactions. There is very easy possible to see, that the considerable most significant is factor A (Infill volume), what is commented above. The second most significant is interaction of factors A and C (Infill volume and Infill type). Then follows factor C (Infill type), Factor B (Layer height). The others interactions are also significant, but with lower weight to measured tensile strength.

There have been made also the measurement with 100% infill volume, just to have complete information about this material. The maximum tensile strength is reached with layer height 0.25 mm, with printing temperature of nozzle 220 °C. And the measured value is 42.18 MPa. Compare this value with the maximum measured value within experiment number 7 (32.10 MPa) it is higher by 10.08 MPa. It is more then 31% difference. So 10% difference in infill volume makes 31% difference in tensile strength in Conductive PLA material.

### 3.2. Evaluation of Resistance Measurement

The second part of this paper and of our experiments is determination of electric resistance of Conductive PLA material, following the prepared design of experiment. For each setup (experiment) 5 samples were measured, for repeatability of measurements and exclusion of random errors. A total of 200 measurements were performed. Measured values are placed to the Table 7, where is also visible the design of experiment for better orientation and recognizing which values belong to which combination of factors and their levels.

**Figure 8:**
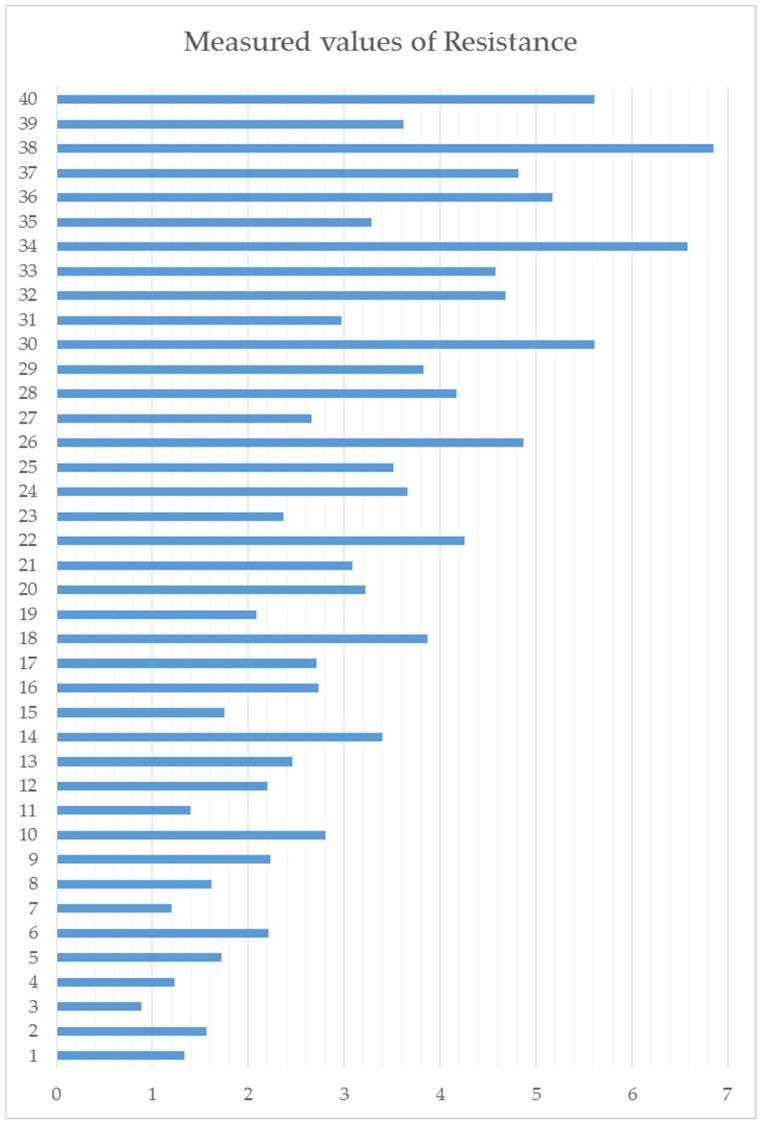
Presentation of measured electrical resistance.

All average values from each measurement and each experiment are also placed to the Figure 8, where are graphically displayed. There is better visualized differences among of single measurements. It can be seen from the graphical presentation, that in certain regular cycles, high values of measured resistance appear. The peaks are for example in experiments 38, 34, 30, 26, 22, 18 and others in the same spacing. The common similarity is in factors B and C, where all of them have nozzle temperature 190 °C and Measurement temperature 80 °C. So this settings is not suitable if it is necessary to reach the lowest resistance. In this case is necessary to increase the printing nozzle temperature and also temperature during the measurement.

To reach the exact information what factors are significant and which are the most significant, the ANOVA statistical method have been executed. Same as in the previous experiments evaluation, the software tool is used for easier and faster evaluation. The final output from the software is visible in below (Table 8). There are mentioned all of the factors and also all interactions. Factor C (Measurement temperature) is evaluated as most significant for resistance. With higher measuring temperature the resistance also higher. This is the negative effect. The second most significant factor is A, what is the length of measured sample. As expected, the resistance is growing by linear way as is defined by known formulas. The third most significant is also factor B (Nozzle temperature). In this case the temperature set during material extrusion have positive effect. It means that with higher temperature is possible to reach produced sample with lower resistance. If we see the interactions of mentioned factors, the next in the row is interaction between factors A and C. The last significant for this experiments is interaction between factors A and B. Others interactions are not significant.

**Figure 9:**
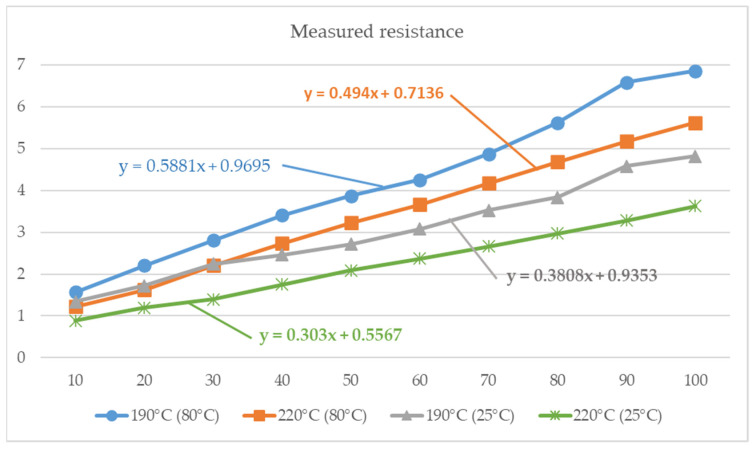
Dependance of resistance on sample length for measurement at 25 °C and 80 °C.

For better visualization there is prepared two figures where are compared measured values. Figure 9 shows the measured values when measured within temperature 25 °C and 80 °C. We can see what is the resistance depending on length of sample. In the figure are two curves, which are comparing this dependence for samples produced with 190 °C and 220 °C of nozzle during production.

In the Figure 9 is possible to see also linear regression model for calculating of resistance for specified properties. This models could be used to calculate resistance also for the situations, where the experiments were not made.

## 4. Conclusions

From the information and results presented above, the following conclusions can be stated. From the previous experiments is known the strength of the classical PLA material at 90% filling of the internal volume of the sample is 48.63 MPa [40]. Similar results are available also from similar research in the range 47 MPa to 53 MPa [41]. The experiment carried out in this paper with Conductive PLA materials shows the highest achieved tensile strength value of 32.1 MPa. This means that Conductive PLA material achieves 66% of the strength of conventional PLA material without additives. The assumption that adding Carbon Black filler to the PLA matrix reduces its strength is confirmed. The results also show how changing the production settings of the parts affects their final strength.

From experiments concerning the measurement of the conductivity resistance of the Conductive PLA material, the following can be determined. The known linear dependence of the resistance on the length of the printed samples was confirmed. It can be seen from the graphs (Figure 9) that if we produce samples with a higher melting temperature of the material during its production, we achieve a lower resistance. On the other hand, if we measure at a higher temperature, the resistance is higher. From the data obtained from an extensive experiment, it was possible to prepare regression calculation models for the calculation of conductivity resistance, without the need for its experimental determination. When comparing our outputs with other research related to electrical resistance of polymers, we can state that the values from other works that are focused on the electrical resistance of polymers are in the range of measured values from our research [42,43,44,45].

## Figures and Tables

**Table 1:** Factors and levels for design of experiment for tensile strength testing.

FACTORS	LEVEL 1	LEVEL 2
A—INFILL VOLUME	50%	90%
B—LAYER HEIGHT	0.125 mm	0.250 mm
C—INFILL TYPE	Rectilinear	Honeycomb

**Table 2:** Design of experiment for tensile strength measurement.

Exp.	A	B	C	A	B	C
1	1	1	1	50%	0.125 mm	Rectilinear
2	1	1	2	50%	0.125 mm	Honeycomb
3	1	2	1	50%	0.250 mm	Rectilinear
4	1	2	2	50%	0.250 mm	Honeycomb
5	2	1	1	90%	0.125 mm	Rectilinear
6	2	1	2	90%	0.125 mm	Honeycomb
7	2	2	1	90%	0.250 mm	Rectilinear
8	2	2	2	90%	0.250 mm	Honeycomb

**Table 3:** Factors and levels for design of experiment for resistance measurement.

Factors	Level 1	Level 2	Level x	Level 10
A—length	10 mm	20 mm	X mm	100 mm
B—Nozzle temperature	190 °C	220 °C	-	-
C—measurement temp.	25 °C	80 °C	-	-

**Table 4:** Design of experiment for resistance measurement.

Exp.	A	B	C	A	B	C
1	1	1	1	10 mm	190 °C	25 °C
2	1	1	2	10 mm	190 °C	80 °C
3	1	2	1	10 mm	220 °C	25 °C
4	1	2	2	10 mm	220 °C	80 °C
5	2	1	1	20 mm	190 °C	25 °C
6	2	1	2	20 mm	190 °C	80 °C
7	2	2	1	20 mm	220 °C	25 °C
8	2	2	2	20 mm	220 °C	80 °C
9	3	1	1	30 mm	190 °C	25 °C
10	3	1	2	30 mm	190 °C	80 °C
11	3	2	1	30 mm	220 °C	25 °C
12	3	2	2	30 mm	220 °C	80 °C
13	4	1	1	40 mm	190 °C	25 °C
14	4	1	2	40 mm	190 °C	80 °C
15	4	2	1	40 mm	220 °C	25 °C
16	4	2	2	40 mm	220 °C	80 °C
17	5	1	1	50 mm	190 °C	25 °C
18	5	1	2	50 mm	190 °C	80 °C
19	5	2	1	50 mm	220 °C	25 °C
20	5	2	2	50 mm	220 °C	80 °C
21	6	1	1	60 mm	190 °C	25 °C
22	6	1	2	60 mm	190 °C	80 °C
23	6	2	1	60 mm	220 °C	25 °C
24	6	2	2	60 mm	220 °C	80 °C
25	7	1	1	70 mm	190 °C	25 °C
26	7	1	2	70 mm	190 °C	80 °C
27	7	2	1	70 mm	220 °C	25 °C
28	7	2	2	70 mm	220 °C	80 °C
29	8	1	1	80 mm	190 °C	25 °C
30	8	1	2	80 mm	190 °C	80 °C
31	8	2	1	80 mm	220 °C	25 °C
32	8	2	2	80 mm	220 °C	80 °C
33	9	1	1	90 mm	190 °C	25 °C
34	9	1	2	90 mm	190 °C	80 °C
35	9	2	1	90 mm	220 °C	25 °C
36	9	2	2	90 mm	220 °C	80 °C
37	10	1	1	100 mm	190 °C	25 °C
38	10	1	2	100 mm	190 °C	80 °C
39	10	2	1	100 mm	220 °C	25 °C
40	10	2	2	100 mm	220 °C	80 °C

**Table 5:** Measured values of Tensile Strength.

Exp.	A	B	C	Rm1	Rm2	Rm3	Rm4	Rm (MPa)
1	1	1	1	12.84	12.84	12.59	13.04	12.83
2	1	1	2	16.47	16.12	17.76	17.76	17.03
3	1	2	1	19.02	18.96	17.84	18.85	18.66
4	1	2	2	18.24	19.1	18.1	18.75	18.55
5	2	1	1	32.88	31.28	31.02	32.03	31.80
6	2	1	2	23.56	23.67	23.79	24.35	23.84
7	2	2	1	32.58	32.03	31.98	31.82	32.10
8	2	2	2	25.2	25.43	24.9	25.62	25.29

**Table 6:** ANOVA statistical evaluation output.

FACTOR/ INTERACTIONS	F (Calculated)	*p* Significancy	SS	MSe	F_tab_ 0.95
A	F (1, 24) = 3641	*p* < 0.000001	SS = 1056.39	MSe = 0.29	4.242
B	F (1, 24) = 143	*p* < 0.000001	SS = 41.45	MSe = 0.29	4.242
C	F (1, 24) = 197	*p* < 0.000001	SS = 57.19	MSe = 0.29	4.242
A × B	F (1, 24) = 54.3	*p* < 0.000001	SS = 15.76	MSe = 0.29	4.242
A × C	F (1, 24) = 613	*p* < 0.000001	SS = 177.76	MSe = 0.29	4.242
B × C	F (1, 24) = 17.4	*p* < 0.000344	SS = 5.04	MSe = 0.29	4.242
A × B × C	F (1, 24) = 51.5	*p* < 0.000001	SS = 14.93	MSe = 0.29	4.242

**Table 7:** Measured values of resistance.

Exp.	A	B	C	R1	R2	R3	R4	R5	R (Ω)
1	1	1	1	1.33	1.34	1.38	1.36	1.27	1.34
2	1	1	2	1.67	1.77	1.57	1.4	1.42	1.57
3	1	2	1	0.83	0.89	0.97	0.88	0.87	0.89
4	1	2	2	1.2	1.24	1.25	1.22	1.22	1.23
5	2	1	1	1.75	1.73	1.74	1.69	1.7	1.72
6	2	1	2	2.22	2.11	2.26	2.22	2.234	2.21
7	2	2	1	1.13	1.24	1.24	1.21	1.16	1.20
8	2	2	2	1.56	1.68	1.66	1.6	1.6	1.62
9	3	1	1	2.23	2.21	2.19	2.3	2.25	2.24
10	3	1	2	2.89	2.71	2.86	2.83	2.75	2.81
11	3	2	1	1.46	1.39	1.41	1.36	1.36	1.40
12	3	2	2	2.18	2.25	2.16	2.23	2.19	2.20
13	4	1	1	2.45	2.42	2.49	2.49	2.43	2.46
14	4	1	2	3.45	3.39	3.38	3.35	3.45	3.40
15	4	2	1	1.68	1.76	1.77	1.77	1.77	1.75
16	4	2	2	2.76	2.81	2.69	2.67	2.75	2.74
17	5	1	1	2.62	2.75	2.69	2.73	2.78	2.71
18	5	1	2	3.85	3.87	3.85	3.89	3.9	3.87
19	5	2	1	2.08	2.09	2.08	2.09	2.11	2.09
20	5	2	2	3.2	3.3	3.19	3.18	3.25	3.22
21	6	1	1	3.02	3.15	3.04	3.13	3.07	3.08
22	6	1	2	4.16	4.27	4.28	4.35	4.22	4.26
23	6	2	1	2.39	2.34	2.37	2.29	2.45	2.37
24	6	2	2	3.62	3.64	3.68	3.64	3.7	3.66
25	7	1	1	3.47	3.51	3.58	3.51	3.51	3.52
26	7	1	2	4.95	4.84	4.81	4.9	4.87	4.87
27	7	2	1	2.71	2.66	2.67	2.65	2.63	2.66
28	7	2	2	4.13	4.15	4.17	4.18	4.23	4.17
29	8	1	1	3.85	3.81	3.85	3.81	3.83	3.83
30	8	1	2	5.55	5.8	5.57	5.64	5.5	5.61
31	8	2	1	3.07	3	2.97	2.91	2.91	2.97
32	8	2	2	4.8	4.62	4.62	4.75	4.62	4.68
33	9	1	1	4.51	4.55	4.68	4.58	4.58	4.58
34	9	1	2	6.55	6.58	6.54	6.68	6.57	6.58
35	9	2	1	3.22	3.39	3.32	3.27	3.22	3.28
36	9	2	2	5.13	5.16	5.25	5.17	5.16	5.17
37	10	1	1	4.86	4.78	4.89	4.82	4.76	4.82
38	10	1	2	6.89	6.84	6.86	6.83	6.85	6.85
39	10	2	1	3.71	3.63	3.74	3.56	3.44	3.62
40	10	2	2	5.6	5.58	5.54	5.74	5.61	5.61

**Table 8:** ANOVA statistical evaluation output.

FACTOR/ INTERACTION	F (Calculated)	*p* Significancy	SS	MSe	F_tab_ 0.95
A	F (9, 160) = 9690	*p* < 0.000001	SS = 322.73	MSe = 0.01	4.444
B	F (1, 160) = 8436	*p* < 0.000001	SS = 31.22	MSe = 0.01	4.444
C	F (1, 160) = 19177	*p* < 0.000001	SS = 70.96	MSe = 0.01	4.444
A × B	F (9, 160) = 118	*p* < 0.000001	SS = 3.91	MSe = 0.01	4.444
A × C	F (9, 160) = 493	*p* < 0.000001	SS = 16.43	MSe = 0.01	4.444
B × C	F (1, 160) = 3.84	*p* < 0.000001	SS = 0.01	MSe = 0.01	4.444
A × B × C	F (9, 160) = 4.35	*p* < 0.000001	SS = 0.14	MSe = 0.01	4.444

## Data Availability

The data are available at Slovak University of Technology in Bratislava.
